# Supplementary material for: Offspring survival changes over generations of captive breeding
Source: Nat Commun. 2021 May 24;12:3045. doi: 10.1038/s41467-021-22631-0 (PMC8144597; doi:10.1038/s41467-021-22631-0)
Supplement: Supplementary file 1 — Descriptions of Additional Supplementary Files [file 41467_2021_22631_MOESM1_ESM.pdf]

Descriptions of Additional Supplementary Files

Supplementary code 1
Description: An RMarkdown script underlying this analysis.

Supplementary data 1
Description: Data underlying the analysis, one row per offspring. Columns include indID (a unique identifier for each animal), litterID (a unique identifier for each litter), Year (year of birth), Species, Scientific.name, BPROGRAM (refers to global region that animal was born in), F (the offspring's inbreeding coefficient), Gen (the offspring's number of generations in captivity), Sire.Gen (the sire's number of generations in captivity), Sire.age.at.breeding (age of the sire when the offspring was born, in days), Sire.F (the sire's inbreeding coefficient), Dam.Gen (the dam's number of generations in captivity), Dam.age.at.breeding (age of the dam when the offspring was born, in days), Dam.F (the dam's inbreeding coefficient) and Survival (1 =offspring survived to age of reproductive maturity, 0 = offspring died before age of reproductive maturity).
