# Supplementary material for: Offspring survival changes over generations of captive breeding
Source: Nat Commun. 2021 May 24;12:3045. doi: 10.1038/s41467-021-22631-0 (PMC8144597; doi:10.1038/s41467-021-22631-0)
Supplement: Supplementary file 2 — Supplementary Information [file 41467_2021_22631_MOESM2_ESM.pdf]

Supplementary Information

Supplementary Table 1: Phylogenetic signal.
Estimates of phylogenetic signal (lambda) for offspring survival to reproductive maturity and for each of the parameters tested, aside from sire age for which the random slopes model did not converge. P-value is calculated from a likelihood ratio test.

	Lambda estimate	P-value (based on LR test)
Survival	7.119×10^{-5}	1
Dam generation	0.3710	0.4996
Sire generation	0.3406	0.7831
Dam age	7.119×10^{-5}	1
Dam f	0.8403	0.0258
Sire f	0.5576	0.1249
Offspring f	0.4531	0.1936

Supplementary Table 2: Modelled results for offspring survival.

a) Results of offspring survival for the sensitivity testing extended dataset analysis (*N* = 37,484 individuals) after model averaging (conditional average). b) Results of offspring survival for the sensitivity testing extended dataset G_{2+} analysis when individuals with one or both wild-born parents are excluded (*N* = 27,734 individuals). Estimates presented are after model averaging (conditional average).

Predictor	Estimate	Adjusted SE	95% CI	RI
a) All offspring				
Intercept	0.5387	0.2039	0.1391, 0.9382	
Dam generation	-0.0367	0.0188	-0.0735, 0.0002	0.70
Sire generation	0.0350	0.0192	-0.0026, 0.0726	0.83
Dam age at breeding	-0.0755	0.0130	-0.1010, -0.0499	1
Sire age at breeding	0.0522	0.0133	0.0261, 0.0782	1
Dam *f*	0.0172	0.0134	-0.0090, 0.0435	0.32
Sire *f*	-0.0073	0.0135	-0.0337, 0.0192	0.21
Offspring *f*	-0.1526	0.0128	-0.1776, -0.1276	1
b) G_{2+}				
Intercept	0.5280	0.1814	0.1725, 0.8834	
Dam generation	-0.0741	0.0211	-0.1155, -0.0328	1
Sire generation	0.0585	0.0205	0.0182, 0.0988	1
Dam age at breeding	-0.1088	0.0148	-0.1378, -0.0797	1
Sire age at breeding	0.0735	0.0148	0.0445, 0.1026	1
Dam *f*	0.0163	0.0150	-0.0132, 0.0457	0.32
Sire *f*	0.0052	0.0152	-0.0245, 0.0349	0.19
Offspring *f*	-0.1884	0.0144	-0.2167, -0.1602	1

Supplementary Table 3: Top model sets.

Top model set (top 2 AIC_C) of generalised linear mixed models for each of the five models where one independent offspring per litter/clutch has been selected. All parameters were retained in each of the five top model sets. Subset 3 was used as a representative model to fit random slopes.

Subset	Model statement	AIC_C	Δ_i[a]	w_i[b]
1	β_0 + Dam age + Sire age + Offspring *f*	27526.9		0.226
	β_0 + Sire generation + Dam age + Sire age + Offspring *f*	27528.1	1.20	0.124
	β_0 + Dam generation + Dam age + Sire age + Offspring *f*	27528.2	1.33	0.116
	β_0 + Dam age + Sire age + Sire *f* + Offspring *f*	27528.6	1.77	0.093
	β_0 + Dam age + Sire age + Dam *f* + Offspring *f*	27528.7	1.81	0.091
2	β_0 + Dam age + Sire age + Offspring *f*	27516.3		0.243
	β_0 + Sire generation + Dam age + Sire age + Offspring *f*	27517.6	1.29	0.127
	β_0 + Dam age + Sire age + Sire *f* + Offspring *f*	27518.3	1.95	0.091
	β_0 + Dam age + Sire age + Dam *f* + Offspring *f*	27518.3	1.97	0.090
	β_0 + Dam generation + Dam age + Sire age + Offspring *f*	27518.3	1.99	0.090
3	β_0 + Dam age + Sire age + Offspring *f*	27527.7		0.224
	β_0 + Sire generation + Dam age + Sire age + Offspring *f*	27528.7	0.99	0.136
	β_0 + Dam generation + Dam age + Sire age + Offspring *f*	27529.3	1.61	0.100
	β_0 + Dam age + Sire age + Dam *f* + Offspring *f*	27529.5	1.81	0.091
	β_0 + Dam age + Sire age + Sire *f* + Offspring *f*	27529.5	1.81	0.090
4	β_0 + Sire generation + Dam age + Sire age + Offspring *f*	27559.7		0.207
	β_0 + Dam age + Sire age + Offspring *f*	27560.7	0.97	0.127
	β_0 + Sire generation + Dam age + Sire age + Sire *f* + Offspring *f*	27561.4	1.69	0.089
	β_0 + Dam age + Sire age + Sire *f* + Offspring *f*	27561.6	1.87	0.081
	β_0 + Dam generation + Sire generation + Dam age + Sire age + Offspring *f*	27561.7	1.96	0.078
5	β_0 + Sire generation + Dam age + Sire age + Offspring *f*	27514.2		0.175
	β_0 + Dam age + Sire age + Offspring *f*	27514.3	0.07	0.169
	β_0 + Dam age + Sire age + Sire *f* + Offspring *f*	27515.6	1.31	0.091
	β_0 + Dam generation + Dam age + Sire age + Offspring *f*	27515.8	1.51	0.082
	β_0 + Sire generation + Dam age + Sire age + Sire *f* + Offspring *f*	27516.0	1.79	0.072
	β_0 + Dam generation + Sire generation + Dam age + Sire age + Offspring *f*	27516.2	1.94	0.066
	β_0 + Sire generation + Dam age + Sire age + Dam *f* + Offspring *f*	27516.2	1.98	0.065

[a] Change in AIC_C from the best model.

[b] Akaike model weight.

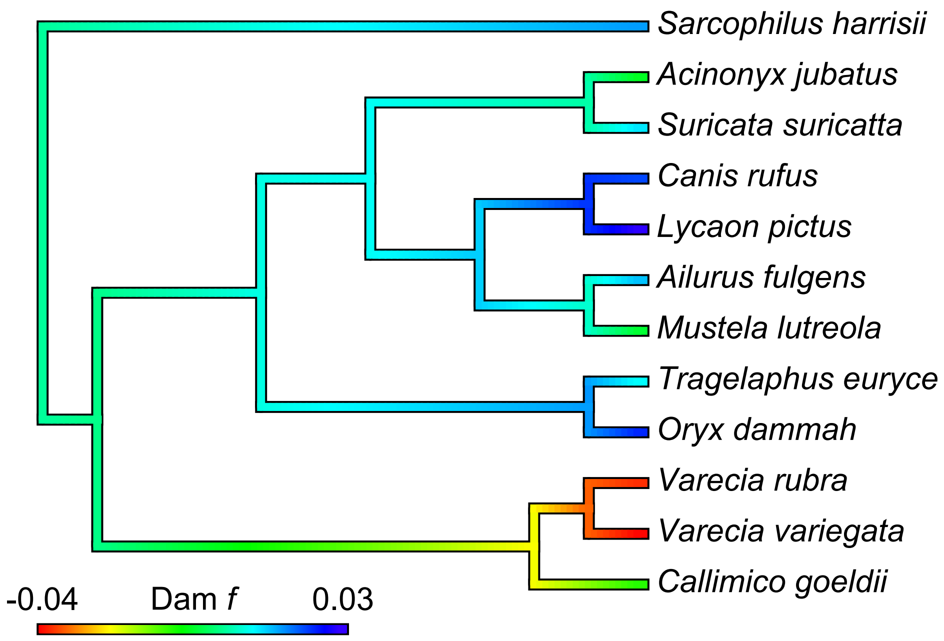

Supplementary Figure 1: Phylogenetic signal of dam *f*
Phylogenetic relationships among the 12 species for which there was enough variation to estimate a random slope for the relationship between dam *f* and offspring survival to reproductive maturity. The tree is shaded by the dam *f* estimate, with a negative (red) estimate indicating a negative impact of dam *f* on offspring survival, and a positive (blue) estimate indicating a positive impact of dam *f* on survival.

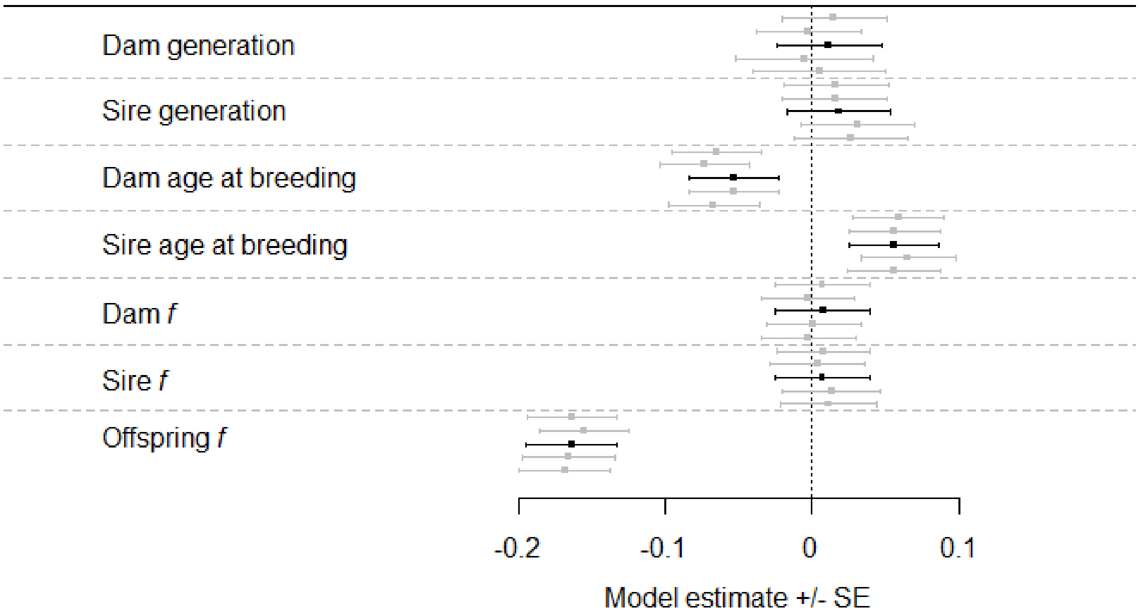

Supplementary Figure 2: Model estimates for offspring survival analysis with five random subsets.

Model estimates (square +/- SE interval) using the five random selections of one independent offspring per litter/clutch (*N* = 21,282 individuals). The third subset (black) was selected as the representative dataset to model random slopes.

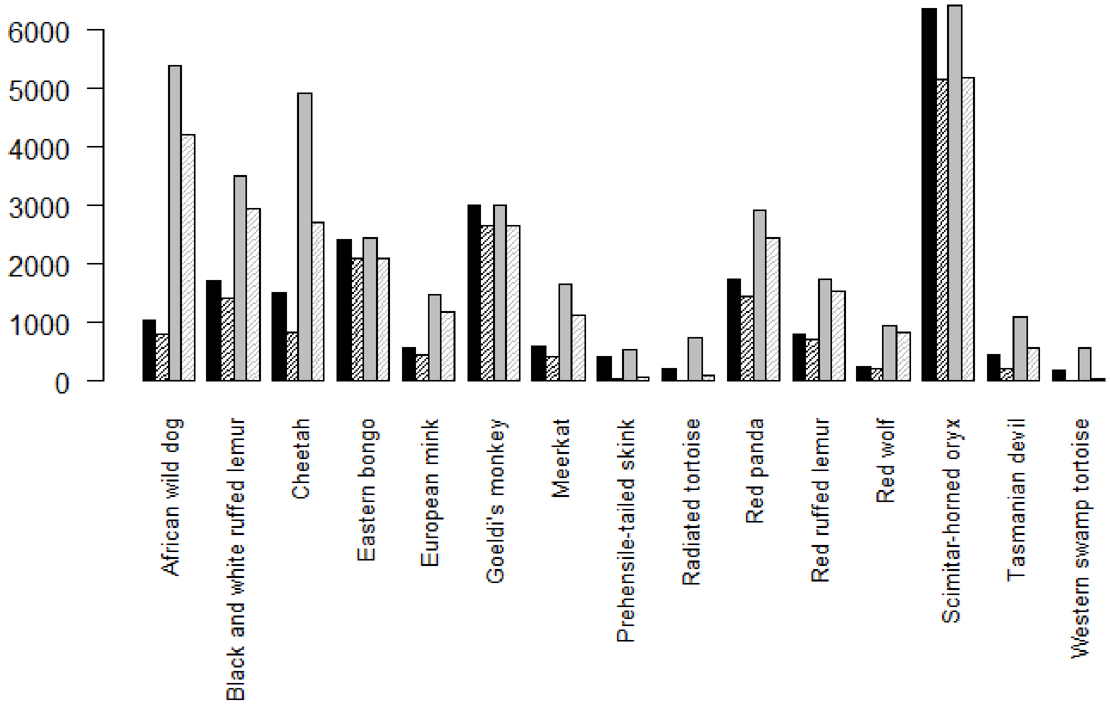

Supplementary Figure 3: Number of individuals in each dataset.

Number of individuals of each species in the main independently sampled litter analysis (black, total *N* = 21,282), independently sampled litter G_{2+} analysis (black hatched, total *N* = 16,516), sensitivity testing extended dataset model (grey, total *N* = 37,484), and sensitivity testing extended dataset G_{2+} model (grey hatched, total *N* = 27,734).

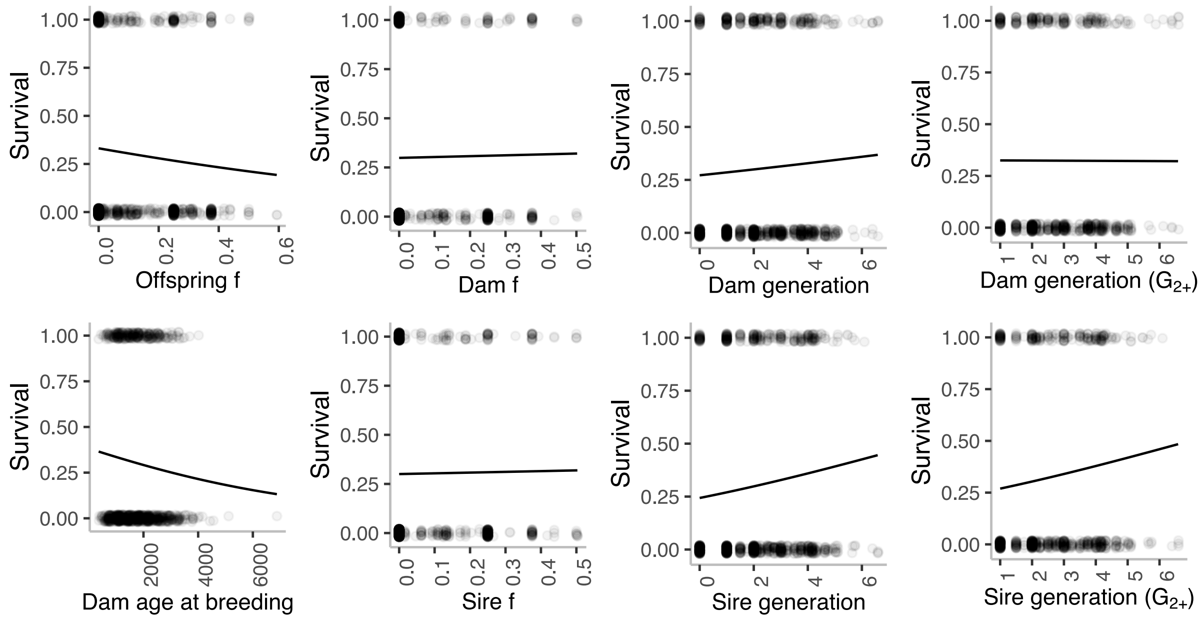

Supplementary Figure 4: Random slope estimates for African wild dog.

Random slopes estimated using a dataset with only one individual per litter/clutch (*N* = 21,282), or the dataset with only one individual per litter/clutch and G_{2+} offspring only (*N* = 16,516) for dam and sire generation G_{2+}. Points represent raw data, shaded by density.

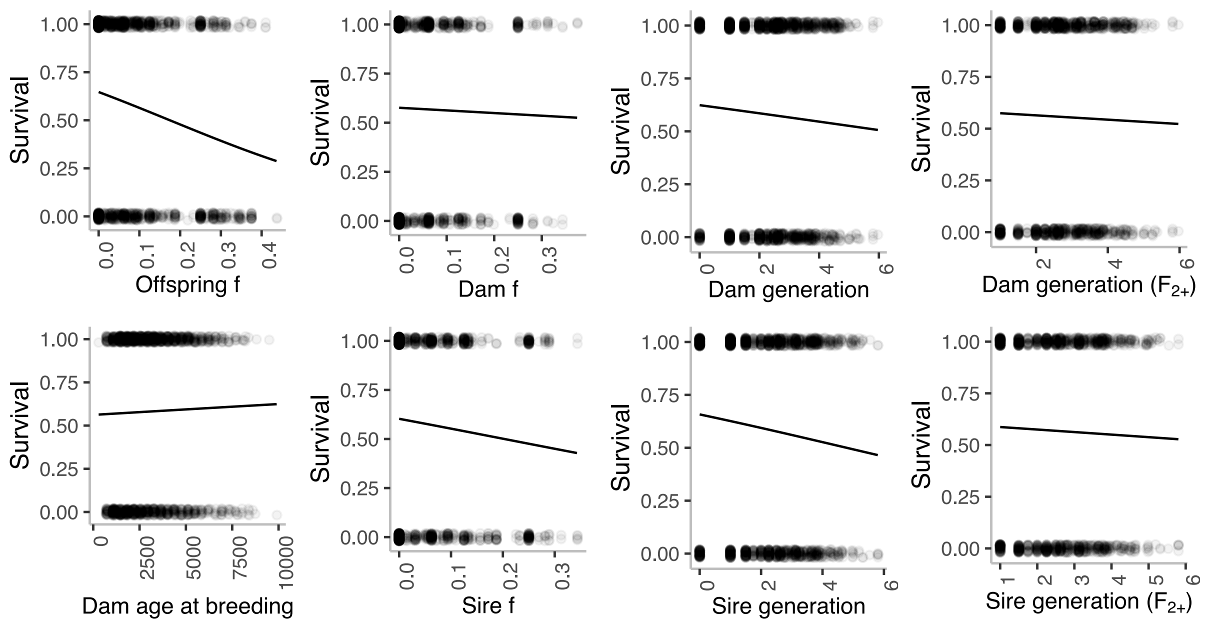

Supplementary Figure 5: Random slope estimates for black-and-white ruffed lemur. Random slopes estimated using a dataset with only one individual per litter/clutch (*N* = 21,282), or the dataset with only one individual per litter/clutch and G_{2+} offspring only (*N* = 16,516) for dam and sire generation G_{2+}. Points represent raw data, shaded by density.

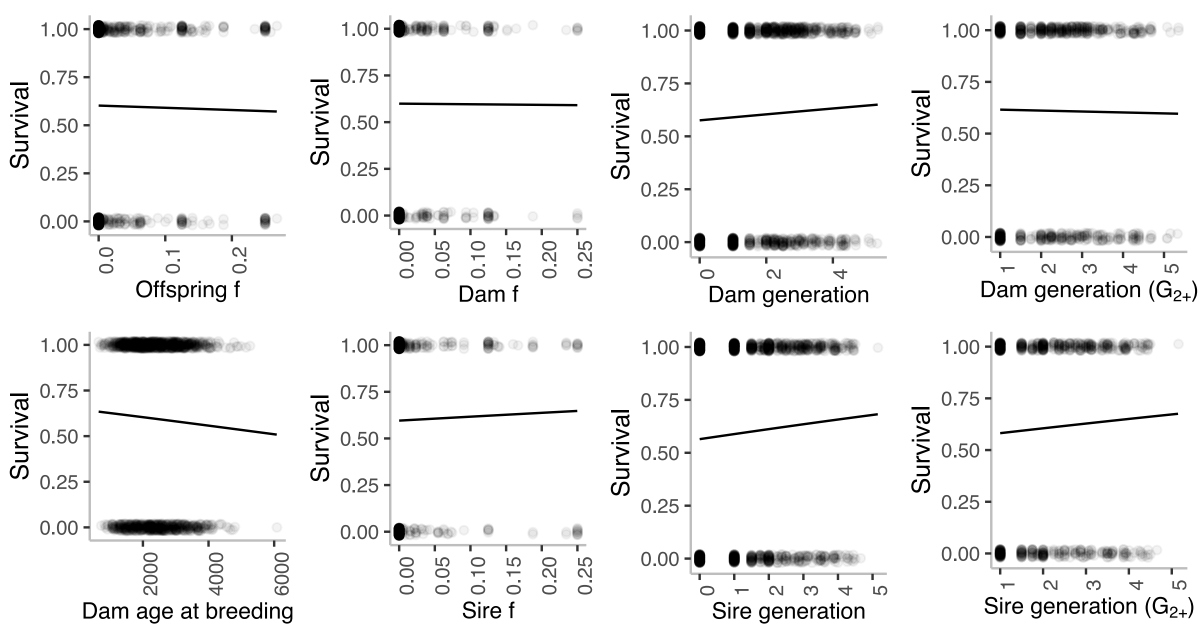

Supplementary Figure 6: Random slope estimates for cheetah.

Random slopes estimated using a dataset with only one individual per litter/clutch (*N* = 21,282), or the dataset with only one individual per litter/clutch and G_{2+} offspring only (*N* = 16,516) for dam and sire generation G_{2+}. Points represent raw data, shaded by density.

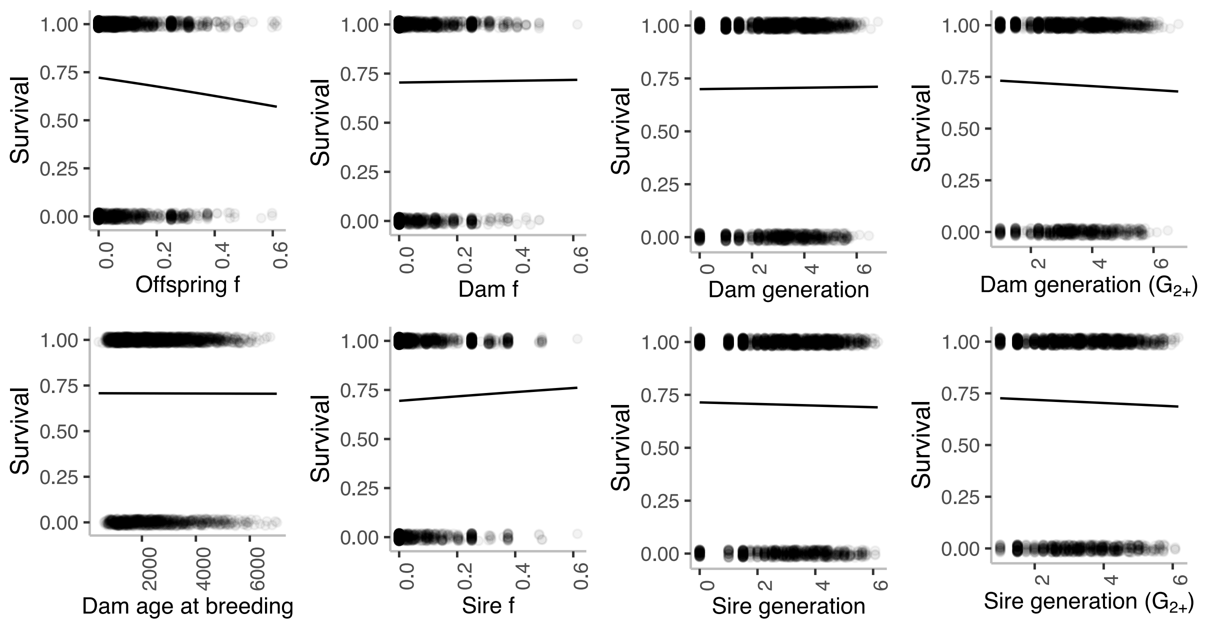

Supplementary Figure 7: Random slope estimates for Eastern bongo.

Random slopes estimated using a dataset with only one individual per litter/clutch (*N* = 21,282), or the dataset with only one individual per litter/clutch and G_{2+} offspring only (*N* = 16,516) for dam and sire generation G_{2+}. Points represent raw data, shaded by density.

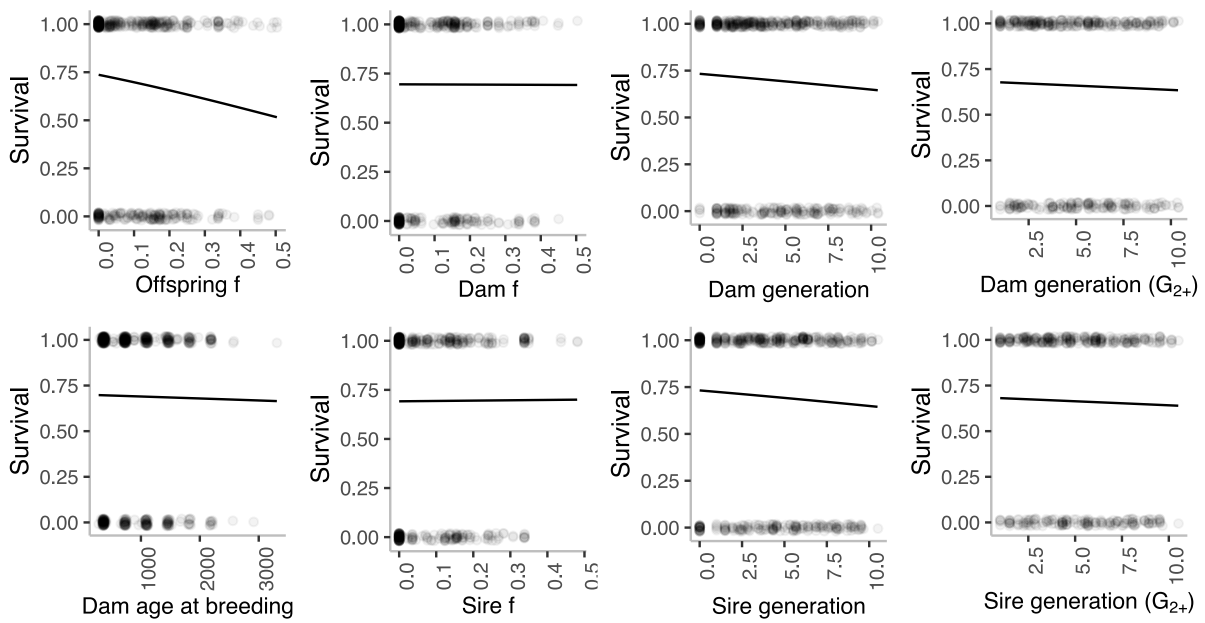

Supplementary Figure 8: Random slope estimates for European mink.

Random slopes estimated using a dataset with only one individual per litter/clutch (N = 21,282), or the dataset with only one individual per litter/clutch and G_{2+} offspring only (N = 16,516) for dam and sire generation G_{2+}. Points represent raw data, shaded by density.

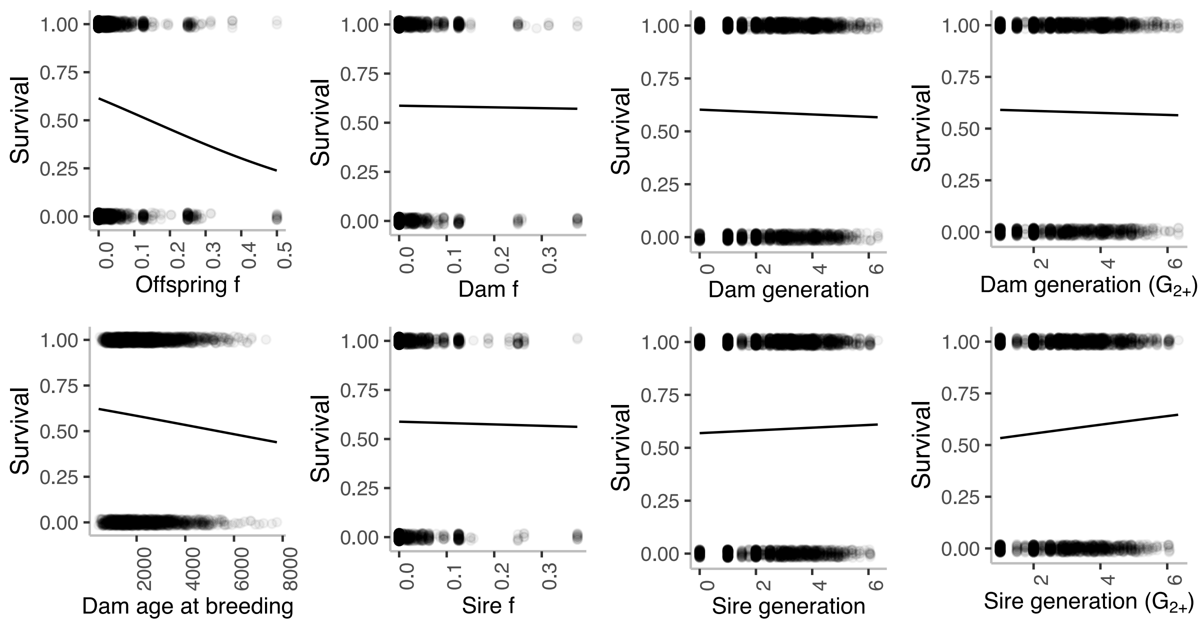

Supplementary Figure 9: Random slope estimates for Goeldi's monkey.

Random slopes estimated using a dataset with only one individual per litter/clutch (*N* = 21,282), or the dataset with only one individual per litter/clutch and G_{2+} offspring only (*N* = 16,516) for dam and sire generation G_{2+}. Points represent raw data, shaded by density.

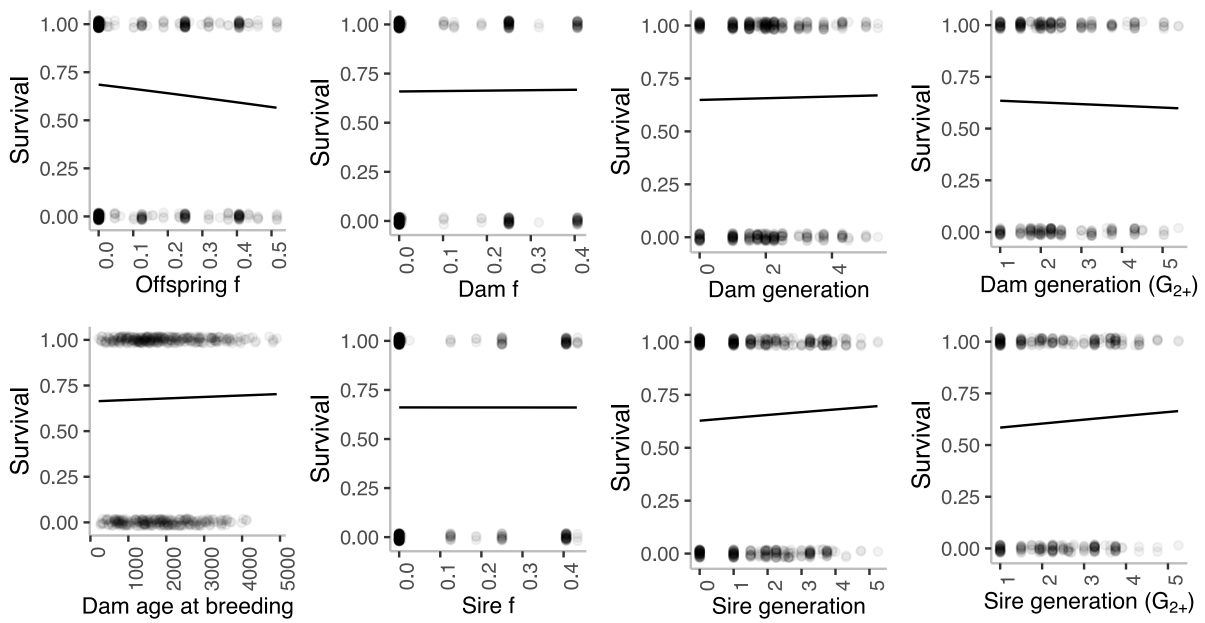

Supplementary Figure 10: Random slope estimates for meerkat.

Random slopes estimated using a dataset with only one individual per litter/clutch (*N* = 21,282), or the dataset with only one individual per litter/clutch and G_{2+} offspring only (*N* = 16,516) for dam and sire generation G_{2+}. Points represent raw data, shaded by density.

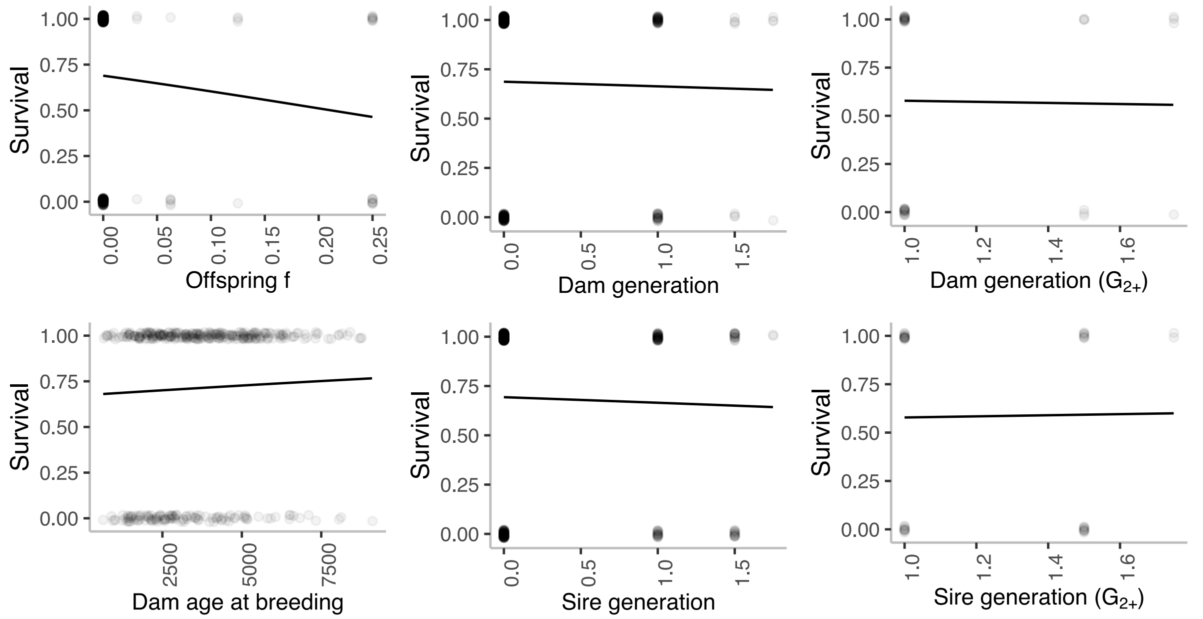

Supplementary Figure 11: Random slope estimates for prehensile-tailed skink.
Random slopes estimated using a dataset with only one individual per litter/clutch (*N* = 21,282), or the dataset with only one individual per litter/clutch and G_{2+} offspring only (*N* = 16,516) for dam and sire generation G_{2+}. Points represent raw data, shaded by density. There was not enough variation in dam or sire inbreeding to fit random slopes for these parameters (Table 1).

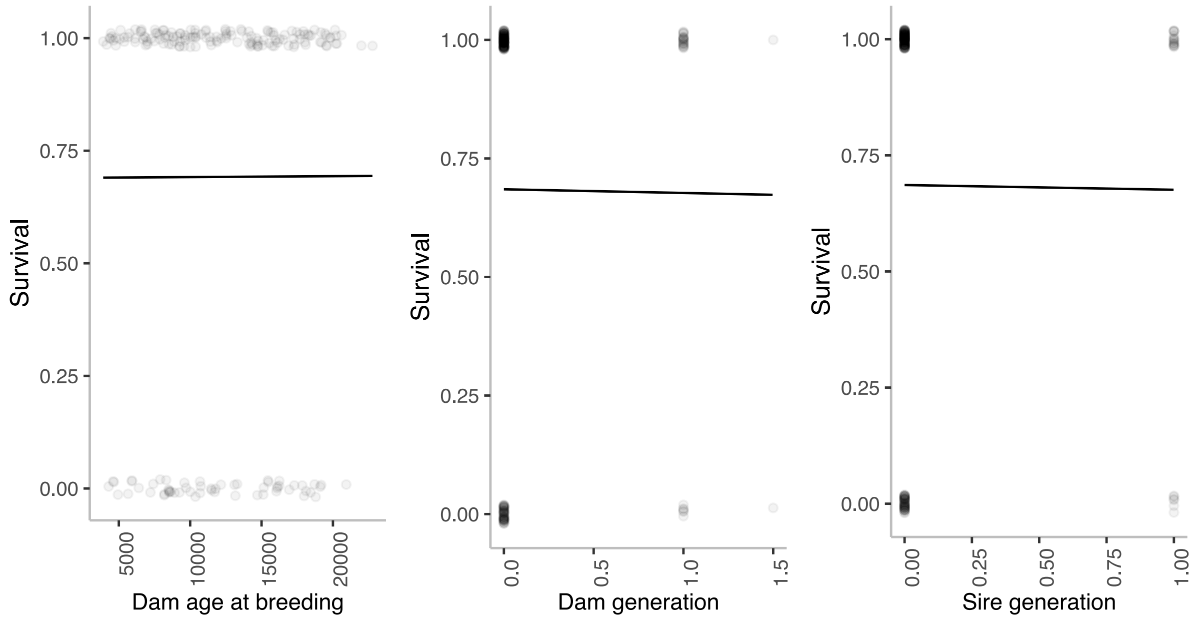

Supplementary Figure 12: Random slope estimates for radiated tortoise.
Random slopes estimated using a dataset with only one individual per litter/clutch (*N* = 21,282). Points represent raw data, shaded by density. There was not enough variation in dam, sire, or offspring inbreeding to fit random slopes for these parameters, nor were there G_{2+} offspring (Table 1).

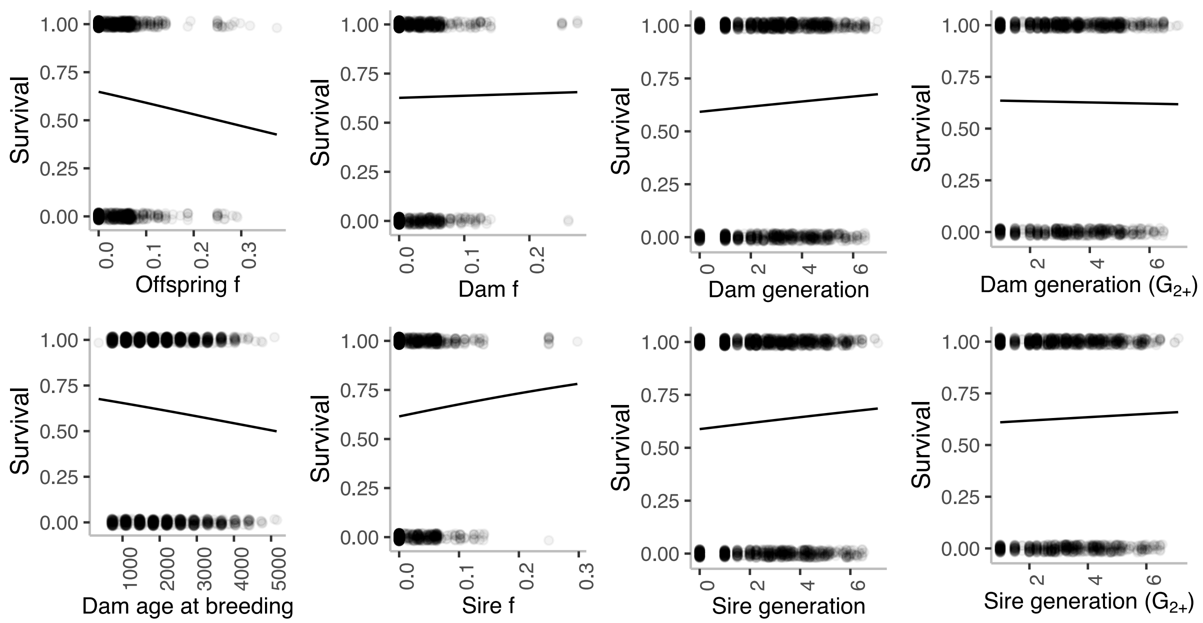

Supplementary Figure 13: Random slope estimates for red panda.

Random slopes estimated using a dataset with only one individual per litter/clutch (*N* = 21,282), or the dataset with only one individual per litter/clutch and G_{2+} offspring only (*N* = 16,516) for dam and sire generation G_{2+}. Points represent raw data, shaded by density.

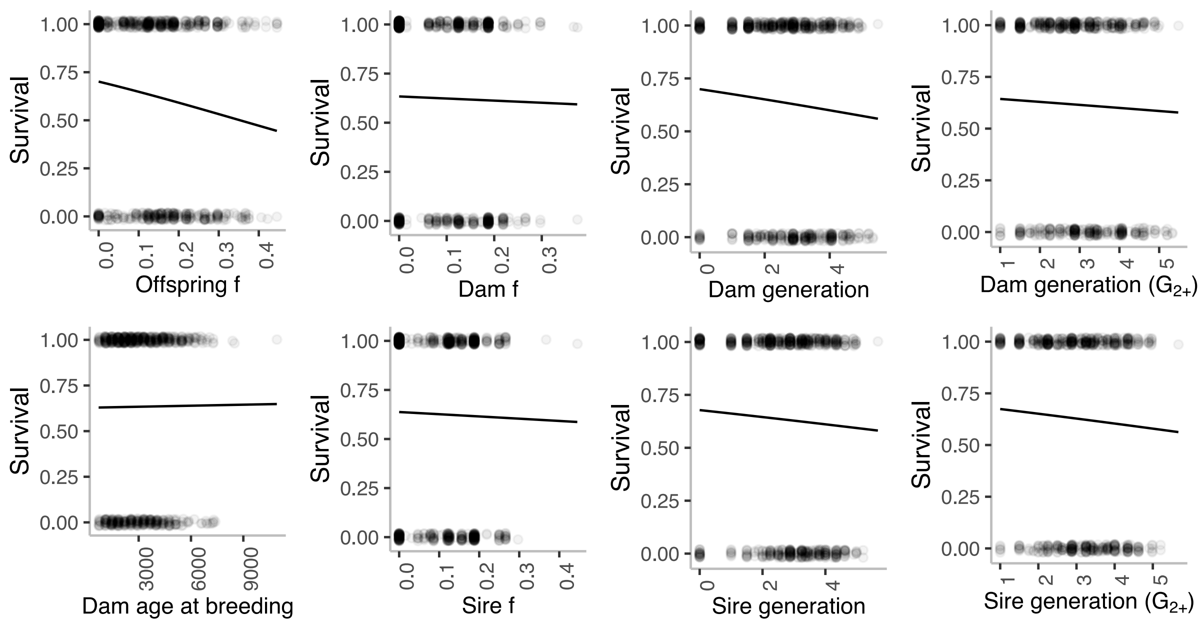

Supplementary Figure 14: Random slope estimates for red ruffed lemur.

Random slopes estimated using a dataset with only one individual per litter/clutch (*N* = 21,282), or the dataset with only one individual per litter/clutch and G_{2+} offspring only (*N* = 16,516) for dam and sire generation G_{2+}. Points represent raw data, shaded by density.

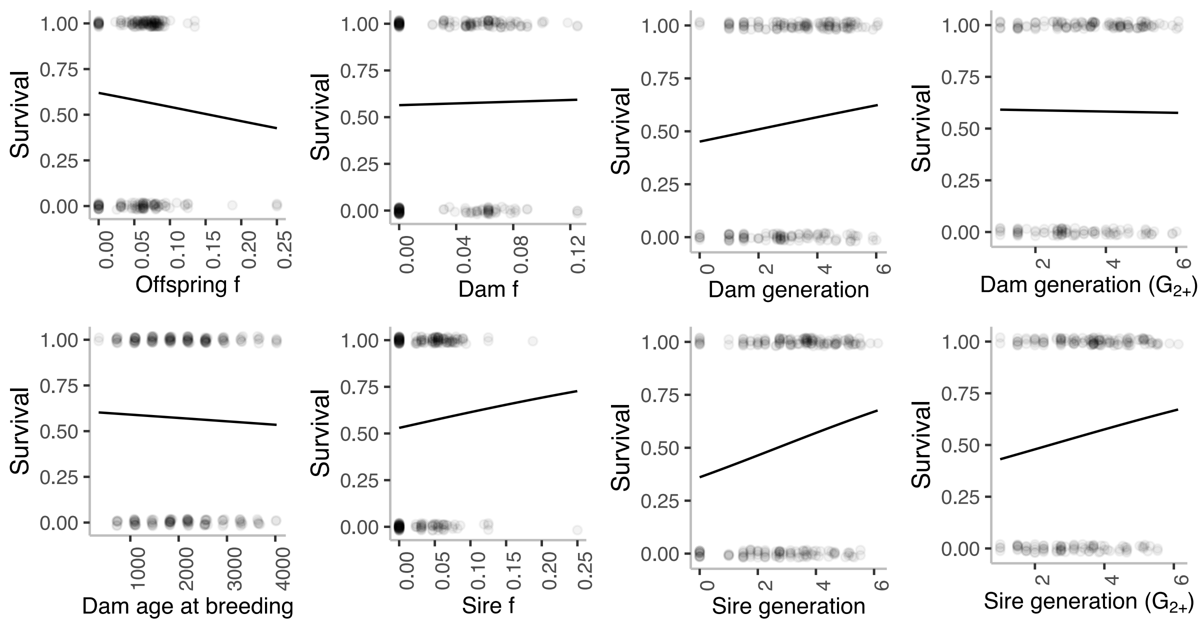

Supplementary Figure 15: Random slope estimates for red wolf.
Random slopes estimated using a dataset with only one individual per litter/clutch (*N* = 21,282), or the dataset with only one individual per litter/clutch and G_{2+} offspring only (*N* = 16,516) for dam and sire generation G_{2+}. Points represent raw data, shaded by density.

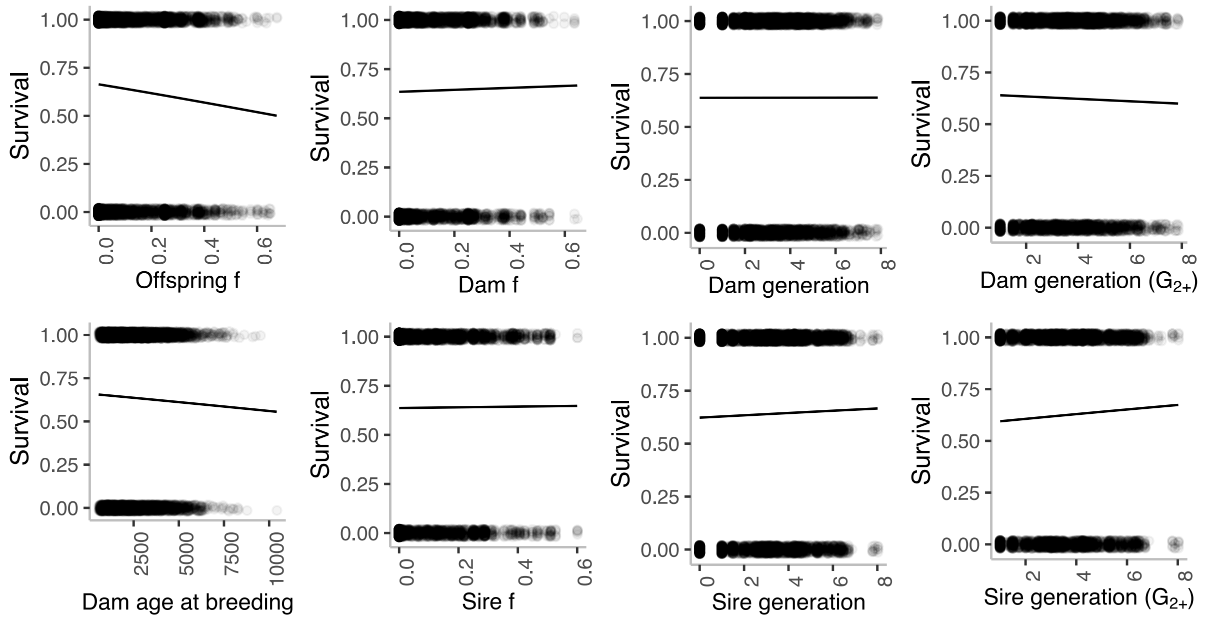

Supplementary Figure 16: Random slope estimates for scimitar-horned oryx.
Random slopes estimated using a dataset with only one individual per litter/clutch (N = 21,282), or the dataset with only one individual per litter/clutch and G_{2+} offspring only (N = 16,516) for dam and sire generation G_{2+}. Points represent raw data, shaded by density.

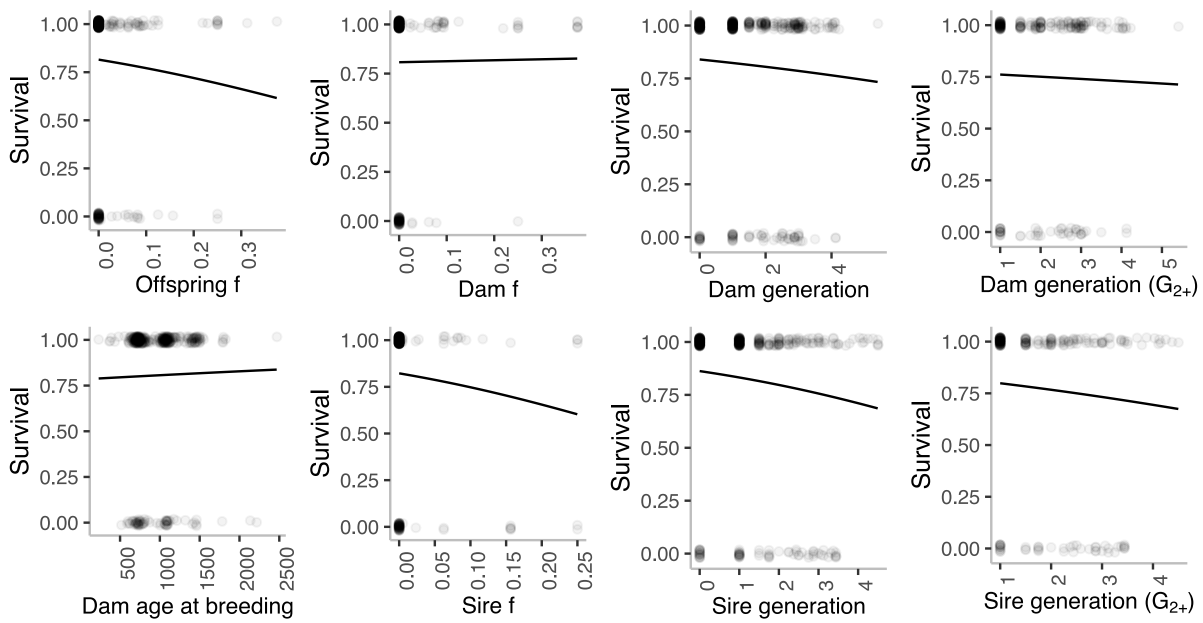

Supplementary Figure 17: Random slope estimates for Tasmanian devil.

Random slopes estimated using a dataset with only one individual per litter/clutch (*N* = 21,282), or the dataset with only one individual per litter/clutch and G_{2+} offspring only (*N* = 16,516) for dam and sire generation G_{2+}. Points represent raw data, shaded by density.

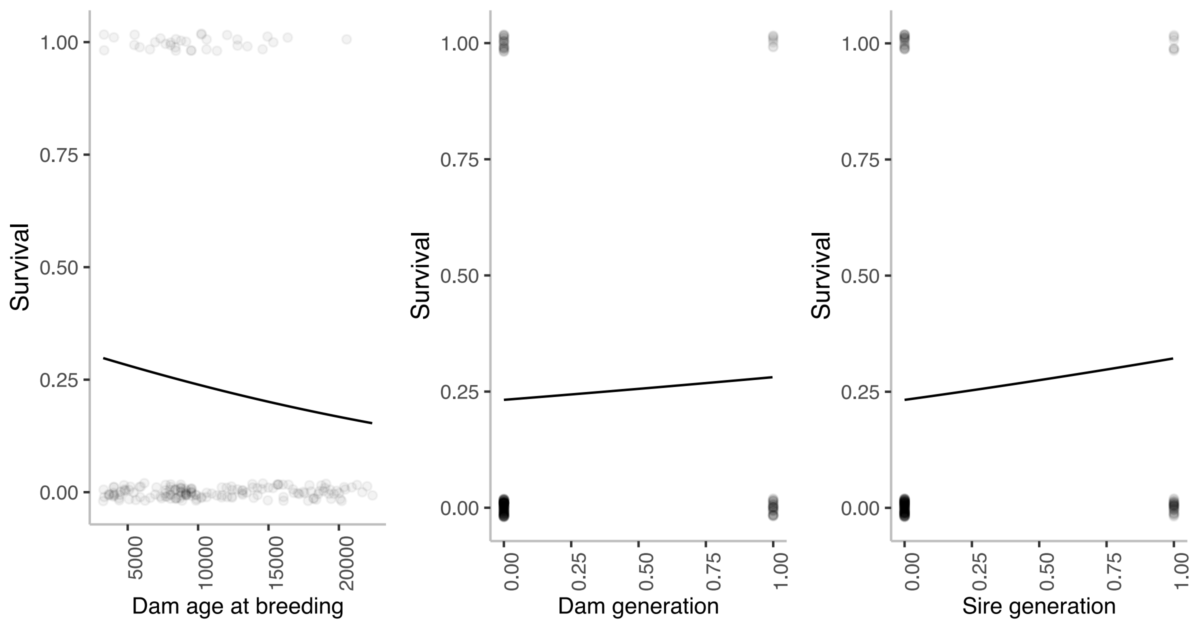

Supplementary Figure 18: Random slope estimates for Western swamp tortoise.
Random slopes estimated using a dataset with only one individual per litter/clutch (*N* = 21,282). There was not enough variation in dam sire or inbreeding to fit random slopes for these parameters, nor were there G_{2+} offspring (Table 1).
