# Supplementary material for: Offspring survival changes over generations of captive breeding
Source: Nat Commun. 2021 May 24;12:3045. doi: 10.1038/s41467-021-22631-0 (PMC8144597; doi:10.1038/s41467-021-22631-0)
Supplement: Supplementary file 6 — Reporting Summary [file 41467_2021_22631_MOESM6_ESM.pdf]

nature research

Corresponding author(s): Catherine Grueber

Last updated by author(s): Feb 26, 2021

Reporting Summary

Nature Research wishes to improve the reproducibility of the work that we publish. This form provides structure for consistency and transparency in reporting. For further information on Nature Research policies, see our Editorial Policies and the Editorial Policy Checklist.

Statistics

For all statistical analyses, confirm that the following items are present in the figure legend, table legend, main text, or Methods section.

n/a	Confirmed	
☐	☒	The exact sample size (*n*) for each experimental group/condition, given as a discrete number and unit of measurement
☐	☒	A statement on whether measurements were taken from distinct samples or whether the same sample was measured repeatedly
☒	☐	The statistical test(s) used AND whether they are one- or two-sided<br>*Only common tests should be described solely by name; describe more complex techniques in the Methods section.*
☐	☒	A description of all covariates tested
☐	☒	A description of any assumptions or corrections, such as tests of normality and adjustment for multiple comparisons
☐	☒	A full description of the statistical parameters including central tendency (e.g. means) or other basic estimates (e.g. regression coefficient) AND variation (e.g. standard deviation) or associated estimates of uncertainty (e.g. confidence intervals)
☐	☒	For null hypothesis testing, the test statistic (e.g. *F*, *t*, *r*) with confidence intervals, effect sizes, degrees of freedom and *P* value noted<br>*Give P values as exact values whenever suitable.*
☒	☐	For Bayesian analysis, information on the choice of priors and Markov chain Monte Carlo settings
☐	☒	For hierarchical and complex designs, identification of the appropriate level for tests and full reporting of outcomes
☒	☐	Estimates of effect sizes (e.g. Cohen's *d*, Pearson's *r*), indicating how they were calculated

Our web collection on statistics for biologists contains articles on many of the points above.

Software and code

Policy information about availability of computer code

Data collection	PMx (freeware; v1.5.8) was used to extract studbook data before processing with custom code. Custom R code underlying this study is available as Supplementary Code 1.
Data analysis	R (version 3.5.1 - 4.0.1, freeware) was used for all data cleaning and analysis. The following R packages were used: dplyr v1.0.0; lme4 v1.1-23; arm v1.11-1; MuMIn v1.43.17; standardize v0.2.1; rotl v3.0.11; ape v5.4; lattice v0.20-41; phytools v0.7-70; ggplot2 v3.3.2; reshape2 v1.4.4; DHARMa v0.3.3.0; metafor v2.4-0; gridExtra v2.3; gplots v3.1.1; performance v0.6.1.

For manuscripts utilizing custom algorithms or software that are central to the research but not yet described in published literature, software must be made available to editors and reviewers. We strongly encourage code deposition in a community repository (e.g. GitHub). See the Nature Research guidelines for submitting code & software for further information.

Data

Policy information about availability of data

All manuscripts must include a data availability statement. This statement should provide the following information, where applicable:

- Accession codes, unique identifiers, or web links for publicly available datasets
- A list of figures that have associated raw data
- A description of any restrictions on data availability

Data underlying this analysis is provided as Supplementary Data 1. All figures can be reproduced using this data and the available code. The Animal Ageing and Longevity (AnAge) Database is available at: https://genomics.senescence.info/species/

Field-specific reporting

Please select the one below that is the best fit for your research. If you are not sure, read the appropriate sections before making your selection.

☐ Life sciences ☐ Behavioural & social sciences ☒ Ecological, evolutionary & environmental sciences

For a reference copy of the document with all sections, see nature.com/documents/nr-reporting-summary-flat.pdf

Ecological, evolutionary & environmental sciences study design

All studies must disclose on these points even when the disclosure is negative.

Study description	The study used existing studbook data from 15 species to investigate factors influencing offspring survival to age of reproductive maturity in captivity. Generalised linear mixed models were used to investigate seven "treatment" factors including dam inbreeding, sire inbreeding, offspring inbreeding, dam generation, sire generation, dam age at breeding and sire age at breeding. A binomial response was fitted where 1= survived, 0 = died. A nested random factor design was applied, to control for differences between Species, Birth Program and Year. Birth Program was nested within Species to account for regional specialisation. Year was also nested within Species to control for improvements in husbandry over the different time frames of the species studied. A total of 37,484 individuals met our criteria for analysis (see exclusions below). We randomly sampled one individual per litter/clutch to avoid issues of statistical non-independence and repeated this process five times to assess reproducibility.
Research sample	Existing studbook data from 15 species managed in captivity was analysed. Studbooks were selected on the basis of availability, size, taxonomic diversity, generations of captive breeding and limited unknown ancestry. Table 1 provides details of the 15 studbooks, including the sample size, effective population size, year of first record, age at maturity, and summary statistics for pedigree inbreeding, generations of captive breeding and age at breeding. The dataset represents the entirety of the managed captive populations of the 15 species included in the analysis.
Sampling strategy	All 58,611 individuals recorded in the 15 studbooks selected were initially considered for analysis. After exclusions (detailed below), 37,484 data points remained. This sample size is sufficient for analysis as it represents the total captive populations of the 15 species included in the analysis.
Data collection	Studbook data was obtained from the relevant studbook keepers that collate it as part of routine management. Studbook data consists of records of individual animals, their date of birth, date of death, parents (if known), and location; and was collected prior to and independently of this study. Permission was sought from the studbook keepers and the relevant regional zoo and aquarium association, and their contributions are appropriately acknowledged.
Timing and spatial scale	This retrospective analysis includes all studbook data collected for the 15 species in the analysis where it met our criteria. The timescale ranged from 1850 to the present, though the timescales of captive breeding differ between species (presented in Table 1 and controlled for in the hierarchical analysis through the nested Species:Year interaction). The spatial scale of each of the studbooks is specified in Table 1 as either international (representing all zoos that breed the species of interest globally) or regional.
Data exclusions	The initial dataset contained 58,611 individuals. Data exclusions include: individuals with unknown parents, all data within the last 364 days of the date of the studbook (to minimise possibility of recent deaths not having been updated), individuals born within the timeframe of the reproductive maturity age (specified in Table 1) from the truncated date (as they would not have the chance to have reached reproductive maturity yet), red wolf animals identified as hybrids in the studbook, and animals born in the wild or released to the wild before the age of reproductive maturity (affected red wolf and Tasmanian devil studbooks). 37,493 remaining individuals had complete data, but 9 outliers were identified and removed, resulting in N = 37,484 individuals for analysis. Missing data appeared random with respect to time.
Reproducibility	Findings were based on retrospective analysis rather than experimental design. As we randomly sampled one offspring per litter/clutch to avoid non-independence issues, we repeated the analysis using five random subsets. Results did not substantially change across the five data subsets (Supplementary Figure 2), and also did not differ substantially from sensitivity testing using the full dataset (without respect to shared litter-mates).
Randomization	Not relevant to study as not an experimental design, therefore there were no treatments to randomize.
Blinding	Not relevant to study as not an experimental design, therefore there were no treatments to blind participants or researchers to.

Did the study involve field work? ☐ Yes ☒ No

Reporting for specific materials, systems and methods

We require information from authors about some types of materials, experimental systems and methods used in many studies. Here, indicate whether each material, system or method listed is relevant to your study. If you are not sure if a list item applies to your research, read the appropriate section before selecting a response.

Materials & experimental systems

n/a	Involved in the study
☒	☐ Antibodies
☒	☐ Eukaryotic cell lines
☒	☐ Palaeontology and archaeology
☒	☐ Animals and other organisms
☒	☐ Human research participants
☒	☐ Clinical data
☒	☐ Dual use research of concern

Methods

n/a	Involved in the study
☒	☐ ChIP-seq
☒	☐ Flow cytometry
☒	☐ MRI-based neuroimaging
